# *In Vitro* Evolution of Cefiderocol Resistance in an NDM-Producing Klebsiella pneumoniae Due to Functional Loss of CirA

**DOI:** 10.1128/Spectrum.01779-21

**Published:** 2021-11-10

**Authors:** Christi L. McElheny, Erin L. Fowler, Alina Iovleva, Ryan K. Shields, Yohei Doi

**Affiliations:** a Division of Infectious Diseases, University of Pittsburgh School of Medicine, Pittsburgh, Pennsylvania, USA; b Center for Innovative Antimicrobial Therapy, University of Pittsburgh School of Medicine, Pittsburgh, Pennsylvania, USA; c Departments of Microbiology and Infectious Diseases, Fujita Health University School of Medicine, Toyoake, Aichi, Japan; Emory University School of Medicine

**Keywords:** iron transporter, siderophore, cefiderocol

## Abstract

By serially exposing an NDM-producing Klebsiella pneumoniae clinical strain to cefiderocol, we obtained a mutant with cefiderocol MIC of >128 μg/ml. The mutant contained an early stop codon in the iron transporter gene *cirA*, and its complementation fully restored susceptibility. The *cirA*-deficient mutant was competed out by the parental strain *in vitro*, suggesting reduced fitness.

**IMPORTANCE** Cefiderocol, a newly approved cephalosporin agent with an extensive spectrum of activity against Gram-negative bacteria, is a siderophore cephalosporin that utilizes iron transporters to access the bacterial periplasm. Loss of functional CirA, an iron transporter, has been associated with cefiderocol resistance. Here, we show that such genetic change can be selected under selective pressure and cause high-level cefiderocol resistance, but with a high fitness cost. Whether these resistant mutants can survive beyond selective pressure will inform stewardship of this agent in the clinic.

## OBSERVATION

Cefiderocol is a catechol-substituted siderophore cephalosporin recently approved for the treatment of complicated urinary tract infection and hospital-acquired and ventilator-associated bacterial pneumonia in the United States and for the treatment of infections due to aerobic Gram-negative bacteria with limited treatment options in the European Union. Cefiderocol has an exceptionally broad spectrum of activity due to its stability against a wide range of beta-lactamases, including metallo-β-lactamases (MBLs), and also because of its active transport across the outer membrane through iron transporter channels (1). Reported mechanisms of resistance to cefiderocol in Gram-negative bacteria include the presence of the β-lactamase genes *bla*_NDM_ and *bla*_PER_ (2). However, production of NDM or PER alone may not be sufficient in causing resistance to cefiderocol. We have previously shown that certain structural changes in AmpC β-lactamases can enable them to hydrolyze cefiderocol, thereby conferring reduced susceptibility or resistance to the agent (3, 4). In addition to the production of β-lactamases, impairment of siderophore uptake systems may also confer resistance to cefiderocol, as has been suggested with Acinetobacter baumannii, where reduced expression or mutation of *pirA* encoding a siderophore receptor has been implicated in the development of cefiderocol resistance (5). Here, we aimed to identify mutations responsible for cefiderocol resistance in Klebsiella pneumoniae.

**FIG 1:**
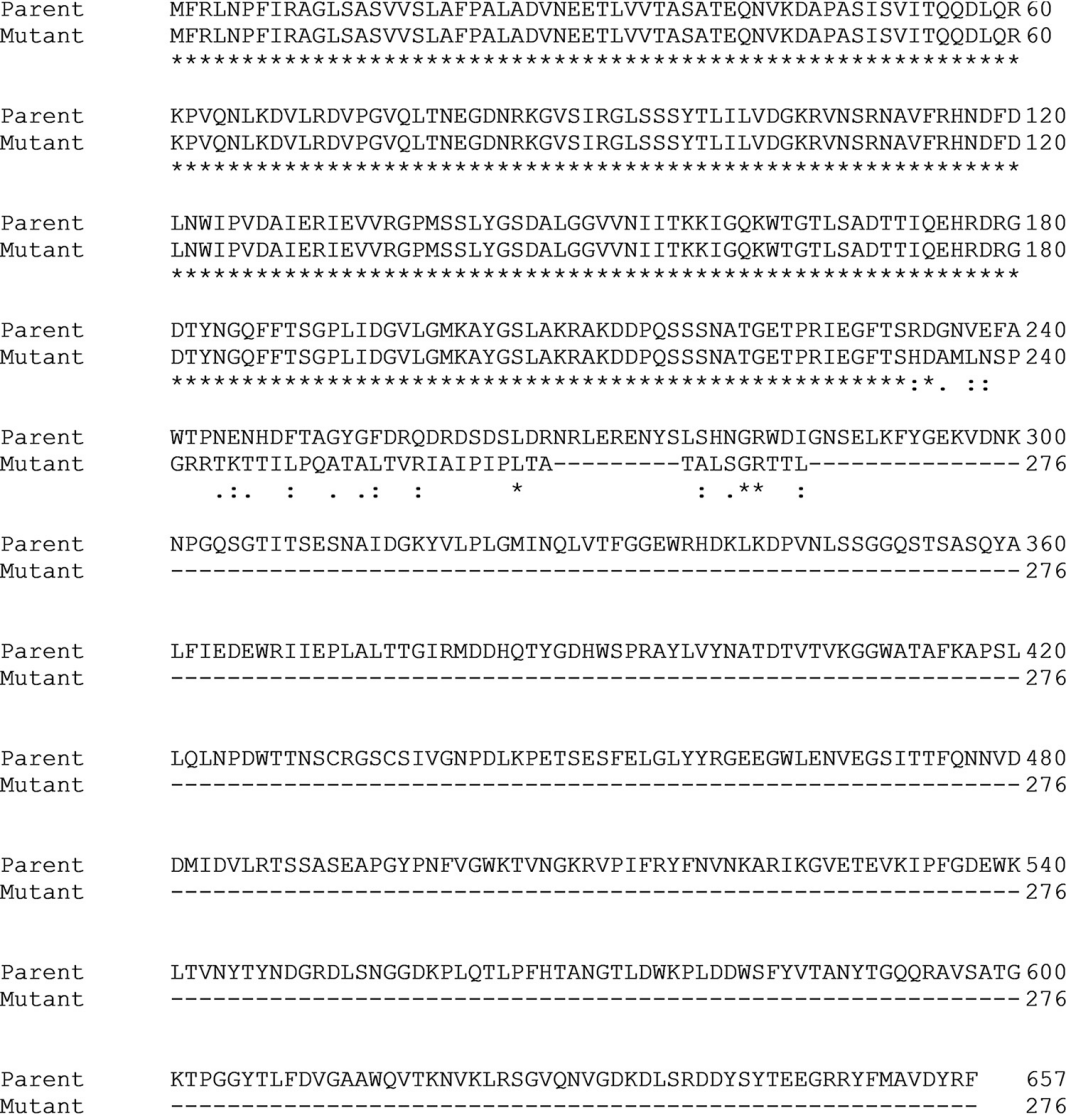
Clustal omega alignment comparing wild-type CirA to the *in vitro* generated mutated CirA. The mutated *cirA* gene has a nucleotide change leading to an R233H substitution and a 1-bp deletion that leads to a frameshift and early stop codon.

A cefiderocol-susceptible NDM-producing K. pneumoniae strain, CNP10 (MIC, 2 μg/ml), was used as a parent strain for the generation of *in vitro* mutants resistant to cefiderocol (6). The strain was serially exposed to increasing concentrations of cefiderocol in cation-adjusted Mueller-Hinton broth overnight with shaking at 37°C. Overnight cultures were transferred daily into fresh media with increasing concentrations of cefiderocol until growth was observed at a concentration of 256 μg/ml. Upon conducting two rounds of the experiment, we obtained one cefiderocol-resistant mutant. This mutant strain had an MIC of >128 μg/ml. Illumina sequencing followed by breseq analysis revealed mutations in the *fabF* (3-oxoacyl-acyl carrier protein synthase II) and *cirA* (iron transporter) genes. The nucleotide change in *fabF* did not lead to an amino acid change. The mutated *cirA* gene had a nucleotide change that caused an R233H substitution and a single base pair deletion that led to a frameshift and early stop codon (Fig. 1).

To define the contribution of the observed deletion to cefiderocol resistance through complementation, a wild-type copy of *cirA* was cloned into shuttle vector pMQ297 (7). Transformants were selected on lysogenic broth agar plates supplemented with hygromycin (140 μg/ml), and the correct sequence was confirmed by Sanger sequencing. The mutant strain was complemented with a wild-type copy of *cirA* on pMQ297. Empty pMQ297 was introduced to the parent CNP10 and cefiderocol-resistant mutant strains as controls. The MICs of CNP10 (pMQ297), *cirA* mutant (pMQ297), and *cirA* mutant (pMQ297-*cirA*) strains were 2 μg/ml, 64 μg/ml, and 2 μg/ml, respectively. Complementation of the *cirA* mutant strain with a wild-type copy of *cirA* therefore fully restored susceptibility to cefiderocol. In a previous report, a mutation of *cirA* and *fiu* in Escherichia coli caused a 16-fold increase in the cefiderocol MIC, whereas deletion of only *cirA* or *fiu* led to a <2-fold increase against the parent strain (8). To our knowledge, this is the first report of genetic changes in a single gene in K. pneumoniae leading to high-level cefiderocol resistance.

To further validate that functional loss of *cirA* causes cefiderocol resistance in K. pneumoniae, we obtained a *cirA* transposon mutant strain (KP07295) along with the parental strain, MKP103, from the Manoil laboratory at the University of Washington. After confirming the disruption of *cirA* through Sanger and whole-genome sequencing, we transformed MKP103 and KP07295 with empty pMQ297 as controls and also transformed KP07295 with pMQ297 carrying wild-type *cirA*. The MICs of MKP103 (pMQ297), KP07295 (pMQ297), and KP07295 (pMQ297-*cirA*) were 0.5 μg/ml, 2 μg/ml, and 0.5 μg/ml, respectively. Again, complementation of the *cirA* mutant strain with a wild-type copy of *cirA* restored the MIC to cefiderocol to the level found in the parent strain, though the fold difference was less than that observed with CNP10. Gram-negative bacteria possess several outer membrane receptors, including FepA, CirA, and Fiu for catecholate-type siderophore transport (9). Thus, the differences in the degree of resistance caused by loss of functional CirA may be due to the relative contribution of each iron acquisition system in a given strain.

We were interested to see if the *cirA* mutations that occurred in CNP10 would affect bacterial fitness, and thus we conducted competitive growth assays. Cultures were grown independently or mixed at a 1:1 ratio in iron-depleted cation-adjusted Mueller-Hinton broth. At 0, 24, 48, and 72 h, 10-fold serial dilutions of each culture were grown on lysogenic broth agar plates with and without hygromycin (140 μg/ml) and cefiderocol (16 μg/ml). The mixed cultures contained CNP10 (pMQ297) and CNP10 *cirA* mutant (pMQ297) or the CNP10 *cirA* mutant (pMQ297) and CNP10 *cirA* mutant (pMQ297-*cirA*). Competitive indexes (CIs) were calculated as follows: the ratio of resistant CFU to susceptible CFU was divided by the ratio of the initial inocula, where competitive index of 1 denotes no change in the ability of either strain to compete. The CIs for the parent and *cirA* mutant strain at 24, 48, and 72 h were 0.0011, 0.0002, and 0.0035, respectively. The competitive indexes for the *cirA* mutant strain and *cirA* complemented strain at 24, 48, and 72 h were 0.0028, 0.0043, and 0.0012, respectively. The competitive indexes were all well below 1, indicating that the mutant strain was readily outcompeted by the wild-type and complemented strains. A growth deficit was previously reported in a *cirA*-deficient *Salmonella* Enteritidis mutant compared to the wild-type grown in M9 medium (10). However, a recent report associated deletions and insertions in *cirA* with *in vivo* development of cefiderocol resistance in a carbapenem-resistant Enterobacter cloacae strain in a patient who had been treated with cefiderocol. The genetic changes in this report led to early stop codons and truncation of CirA, which is similar to the observations in our study. This suggests that such resistant mutants may still predominate in the presence of selective pressure (11).

In conclusion, we report a K. pneumoniae strain with an amino acid substitution and a single base deletion in *cirA* leading to high-level cefiderocol resistance and reduced fitness. The recent report of a CirA-deficient, cefiderocol-resistant E. cloacae strain emerging in a patient during cefiderocol treatment underscores the potential clinical relevance of this resistance mechanism in *Enterobacterales*. Further studies are required to examine genetic changes that may occur as a consequence of clinical use of the agent and their impact on resistance and virulence.

## Supplementary Material

Reviewer comments

## References

[1] Yamano Y. 2019. *In vitro* activity of cefiderocol against a broad range of clinically important Gram-negative bacteria. Clin Infect Dis 69:S544–S551. doi:10.1093/cid/ciz827. 31724049 PMC6853761

[2] Kohira N, Hackel MA, Ishioka Y, Kuroiwa M, Sahm DF, Sato T, Maki H, Yamano Y. 2020. Reduced susceptibility mechanism to cefiderocol, a siderophore cephalosporin, among clinical isolates from a global surveillance programme (SIDERO-WT-2014). J Glob Antimicrob Resist 22:738–741. doi:10.1016/j.jgar.2020.07.009. 32702396

[3] Shields RK, Iovleva A, Kline EG, Kawai A, McElheny CL, Doi Y. 2020. Clinical evolution of AmpC-mediated ceftazidime-avibactam and cefiderocol resistance in *Enterobacter cloacae* complex following exposure to cefepime. Clin Infect Dis 71:2713–2716. doi:10.1093/cid/ciaa355. 32236408 PMC7744991

[4] Kawai A, McElheny CL, Iovleva A, Kline EG, Sluis-Cremer N, Shields RK, Doi Y. 2020. Structural basis of reduced susceptibility to ceftazidime-avibactam and cefiderocol in *Enterobacter cloacae* due to AmpC R2 loop deletion. Antimicrob Agents Chemother 64:e00198-20. doi:10.1128/AAC.00198-20. 32284381 PMC7318025

[5] Malik S, Kaminski M, Landman D, Quale J. 2020. Cefiderocol resistance in *Acinetobacter baumannii*: roles of b-lactamases, siderophore receptors, and penicillin binding protein 3. Antimicrob Agents Chemother 64:e01221-20. doi:10.1128/AAC.01221-20. 32868330 PMC7577126

[6] Lee C-S, Vasoo S, Hu F, Patel R, Doi Y. 2014. *Klebsiella pneumoniae* ST147 coproducing NDM-7 carbapenemase and RmtF 16S rRNA methyltransferase in Minnesota. J Clin Microbiol 52:4109–4110. doi:10.1128/JCM.01404-14. 25143576 PMC4313265

[7] Kalivoda EJ, Horzempa J, Stella NA, Sadaf A, Kowalski RP, Nau GJ, Shanks RM. 2011. New vector tools with a hygromycin resistance marker for use with opportunistic pathogens. Mol Biotechnol 48:7–14. doi:10.1007/s12033-010-9342-x. 20972648 PMC3617578

[8] Ito A, Sato T, Ota M, Takemura M, Nishikawa T, Toba S, Kohira N, Miyagawa S, Ishibashi N, Matsumoto S, Nakamura R, Tsuji M, Yamano Y. 2018. *In vitro* antibacterial properties of cefiderocol, a novel siderophore cephalosporin, against Gram-negative bacteria. Antimicrob Agents Chemother 62:e01454-17. doi:10.1128/AAC.01454-17. PMC5740388 29061741

[9] Miethke M, Marahiel MA. 2007. Siderophore-based iron acquisition and pathogen control. Microbiol Mol Biol Rev 71:413–451. doi:10.1128/MMBR.00012-07. 17804665 PMC2168645

[10] Zhang Z, Du W, Wang M, Li Y, Su S, Wu T, Kang Y, Shan X, Shi Q, Zhu G. 2020. Contribution of the colicin receptor CirA to biofilm formation, antibotic resistance, and pathogenicity of *Salmonella* Enteritidis. J Basic Microbiol 60:72–81. doi:10.1002/jobm.201900418. 31737922

[11] Klein S, Boutin S, Kocer K, Fiedler MO, Storzinger D, Weigand MA, Tan B, Richter D, Rupp C, Mieth M, Mehrabi A, Hackert T, Zimmermann S, Heeg K, Nurjadi D. 2021. Rapid development of cefiderocol resistance in carbapenem-resistant *Enterobacter cloacae* during therapy is associated with heterogeneous mutations in the catecholate siderophore receptor *cirA*. Clin Infect Dis. doi:10.1093/cid/ciab511. PMC8906715 34079986

