# Supplementary material for: In Vitro Evolution of Cefiderocol Resistance in an NDM-Producing Klebsiella pneumoniae Due to Functional Loss of CirA
Source: Microbiol Spectr. 2021 Nov 10;9(3):e01779-21. doi: 10.1128/Spectrum.01779-21 (PMC8579844; doi:10.1128/Spectrum.01779-21)
Supplement: Reviewer comments [file reviewer-comments.pdf]

AMERICAN SOCIETY FOR MICROBIOLOGY | Microbiology Spectrum

In vitro evolution of cefiderocol resistance in an NDM-producing *Klebsiella pneumoniae* due to functional loss of CirA

Christi McElheny, Erin Fowler, Alina Iovleva, Ryan Shields, and Yohei Doi

Corresponding Author(s): Yohei Doi, University of Pittsburgh School of Medicine

Review Timeline:

Submission Date:	October 6, 2021
Accepted:	October 10, 2021

Editor: Joanna Goldberg

Reviewer(s): The reviewers have opted to remain anonymous.

Transaction Report:

(Note: With the exception of the correction of typographical or spelling errors that could be a source of ambiguity, letters and reports are not edited. The original formatting of letters and referee reports may not be reflected in this compilation.)

DOI: https://doi.org/10.1128/Spectrum.01779-21

October 10, 2021

Dr. Yohei Doi
University of Pittsburgh School of Medicine
Division of Infectious Diseases
3550 Terrace Street
Scaife Hall S829
Pittsburgh, PA 15261

Re: Spectrum01779-21 (*In vitro* evolution of cefiderocol resistance in an NDM-producing *Klebsiella pneumoniae* due to functional loss of CirA)

Dear Dr. Yohei Doi:

Your manuscript has been accepted, and I am forwarding it to the ASM Journals Department for publication. You will be notified when your proofs are ready to be viewed.

The ASM Journals program strives for constant improvement in our submission and publication process. Please tell us how we can improve your experience by taking this quick Author Survey.

As an open-access publication, Spectrum receives no financial support from paid subscriptions and depends on authors' prompt payment of publication fees as soon as their articles are accepted. You will be contacted separately about payment when the proofs are issued; please follow the instructions in that e-mail. Arrangements for payment must be made before your article is published. For a complete list of **Publication Fees**, including supplemental material costs, please visit our website.

Corresponding authors may join or renew ASM membership to obtain discounts on publication fees. Need to upgrade your membership level? Please contact Customer Service at Service@asmusa.org.

Thank you for submitting your paper to Spectrum.

Sincerely,

Joanna Goldberg
Editor, Microbiology Spectrum

Journals Department
American Society for Microbiology
1752 N St., NW
Washington, DC 20036
E-mail: spectrum@asmusa.org
